# Measurements of carotid intima media thickness in non-invasive high-frequency ultrasound images: the effect of dynamic range setting

**DOI:** 10.1186/1476-7120-13-5

**Published:** 2015-01-27

**Authors:** Mario Gaarder, Therese Seierstad

**Affiliations:** Department of Radiology and Nuclear Medicine, Oslo University Hospital, P.O. Box 4950, 0424 Nydalen, Oslo, Norway; Faculty of Clinical Medicine, University of Oslo, P.O. Box 1078, 0316 Blindern, Oslo, Norway

**Keywords:** Carotid intima media thickness, Dynamic range, Ultrasound, Carotid arteries

## Abstract

**Background:**

Carotid intima media thickness (CIMT) measured with ultrasound (US) is widely used as biomarker for arteriosclerosis and as surrogate endpoint in interventional studies to assess efficacy of drug therapies. Strict US protocols are necessary to ensure reproducibility. The range of US signal intensities used for image formation, the dynamic range (DR), is rarely reported in studies and little is known about its effect on CIMT measurements in humans. The purpose of this study was to quantify the impact of DR on measurements of CIMT.

**Methods:**

US was used to examine 313 carotid arteries in participants from two different clinical studies. For each artery, images with DR of 40, 55, 70 and 85 dB were captured from the same frozen US frame. Mean CIMT (CIMT_mean_), maximum CIMT (CIMT_max_) and standard deviation of CIMT (CIMT_sd_) were obtained for all images. CIMT for different DRs were compared using student *t*-test.

**Results:**

CIMT_mean_ for 40, 55, 70 and 85 dB were 0.529, 0.564, 0.590 and 0.605 mm respectively. For CIMT_max_ the corresponding values were 0.626, 0.667, 0.698, and 0.716 mm. CIMT_mean_ and CIMT_max_ increased significantly for increasing DR steps (p < 0.01). The relative change in CIMT_mean_ and CIMT_max_ were largest between 40 and 55 dB (6.7% and 7.0%) and smallest between 70 and 85 dB (2.6% and 2.7%) indicating a declining dependency for increasing DR.

**Conclusions:**

DR significantly changes CIMT measurements and the changes are most prominent for lower DRs. The effect of changing DR is larger in human arteries than in phantoms. Reporting the DR will therefore increase the validity of CIMT data.

## Background

Ultrasound (US) measurements of carotid intima-media thickness (CIMT) are used to assess the extent of arteriosclerosis and are a biomarker for the future risk of cardiovascular disease (CVD) and stroke [1–6]. Rate of CIMT change is also used as a surrogate endpoint for CVD in interventional studies to assess early effects of drug therapies [7, 8].

**Figure 1:**
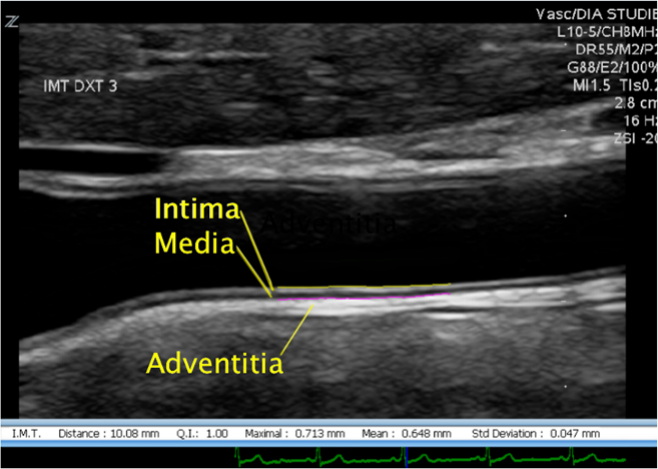
**Intima-media complex in the CCA.** The figure shows the distal two centimetres of the CCA. Intima, media and adventitia are indicated as well as the one centimetre area in which CIMT is measured.

In US images the carotid artery wall is depicted as three separate layers: intima, media and adventitia (Figure 1). The CIMT is defined as the distance from the lumen-intima interface to the media-adventitia interface [9]. The intima-media complex can easily be distinguished from the surrounding tissue in US images and the distinct borders allows for manual as well as automatic measurements of the CIMT.

Reproducibility is a prerequisite for a reliable biomarker, and standardization of imaging and measurement of CIMT will minimize variability between measurements and between readers. Development of US protocols for measuring CIMT has been focusing on choice of vessel segment, near or far wall measurements, and frequency setting as well as performance of the technicians and the observers [5]. The updated Mannheim Carotid Intima-Media Thickness and Plaque Consensus [5] suggests that the dynamic range (DR) should be set at approximately 60 decibel (dB). The DR defines the number of US echo intensities included in the image formation process. Increased DR will increase the range of signal intensities resulting in a low-contrast image. Oppositely, decreased DR will decrease the range of signal intensities resulting in a high-contrast image.

It has previously been shown that change in DR has a significant effect on measurements of wall thickness and lumen diameter in phantoms [10], however the documentation on effects in human arteries are lacking..

The purpose of this study was to quantify the effects of changing DR in non-invasive US CIMT measurements in the common carotid arteries (CCA).

## Methods

### Study population

From August 2012 to January 2014, 165 participants were included in this prospective study. The participants were recruited from the two clinical studies *Arteriosclerosis and childhood diabetes* (the ABD-study, n = 96) and *Impact of insulin sensitivity on cardiovascular risk markers during 9–19 years of follow-up* (the INFO-study, n = 69). The characteristics of the populations are presented in Table 1. The Regional Committee for Medical and Health Research Ethics South East approved both studies (ABD (REC ID 2011/1818), INFO (REC ID 2010/3339)) and written informed consent was obtained from all participants.

**Table 1:** **Demographic data**

	Total	The ABD-study	The INFO-study
Arteries [n]	313	181*	132**
Participants [n]	165	96	69
Males [%]	31	47	100
Age [yrs.]	27.7 (10.9)	19.0 (3.1)	39.9 (3.5)
Diabetics [%]	31	60	1
Systolic BP [mmHg]	123.6 (11.1)	119.6 (9.5)	129.2 (10.7)
Diastolic BP [mmHg]	74.5 (9.5)	69.9 (7.6)	81.0 (8.1)

*missing DR 40 dB data in one participant, **missing DR 40 dB data in two participants, and 55, 70 and 85 dB data in one participant. Only one artery was examined in nine participants in the ABD-study and in five participants in the INFO-study.

### US examination

All US examinations were performed using Zonare Z-One ULTRA US equipment (Zonare Medical Systems, Mountain View, CA, USA) equipped with a linear 10–5 transducer and a three lead electrocardiogram (ECG). A frequency of 8 MHz and its harmonic frequencies were used. All examinations were performed using the same protocol (compound imaging, edge enhancement and standard B-mode grey scale images) and by the same operator (MG). The study participants were instructed to abstain from alcohol, caffeine and nicotine 12 hours prior to the examination and to only consume clear liquids on the morning of the examination.

All participants were examined in the supine position with the neck extended and the head tilted slightly towards the opposite of the examined side. Both left and right CCAs were depicted. The three ECG pads were attached to both shoulders and the left hip prior to US examination, and brachial blood pressure was measured after at least ten minutes in the supine position.

**Figure 2:**
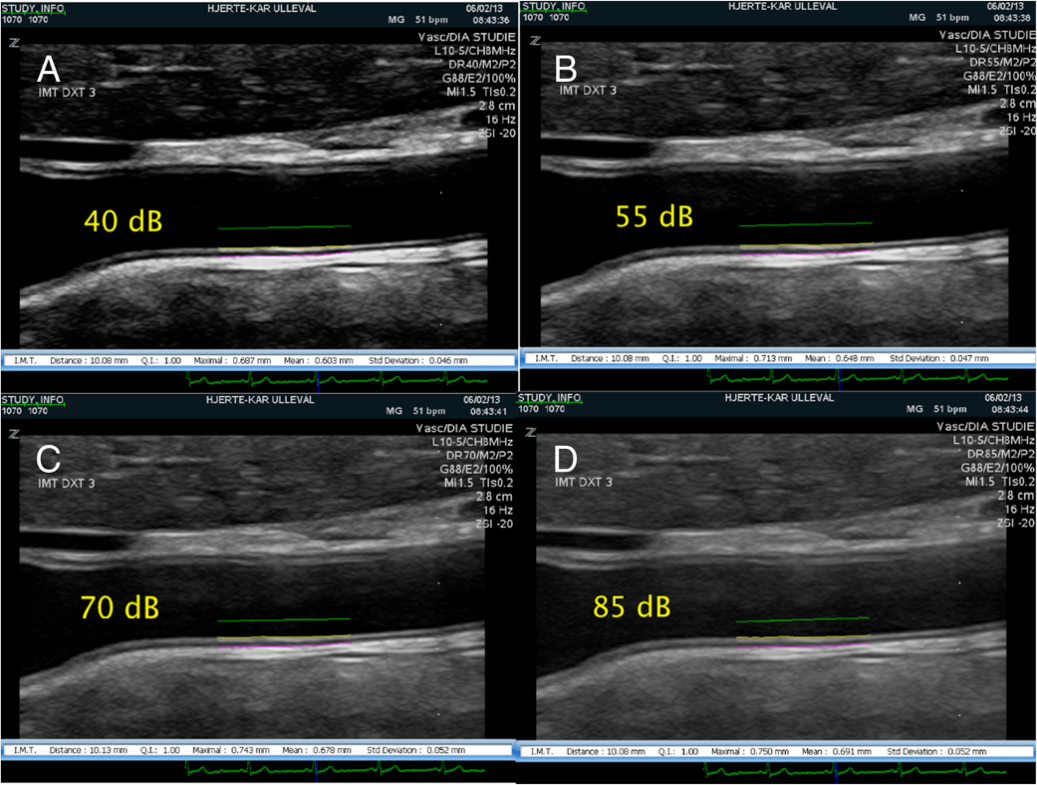
**CIMT measurements of the same CCA for different DRs.** The figure show from top left (clockwise) identical US images of the CCA, where the DR is changed, and the CIMT_mean_ and CIMT_max_ measurements differ. **A)** This image is captured using DR 40 dB, yielding CIMT_mean_ and CIMT_max_ measurements of 0.603 and 0.687 millimetres, respectively **B)** US image captured using 55 dB, CIMT_mean_ and CIMT_max_ were 0.648 and 0.713 millimetres. **C)** US image captured using 70 dB, CIMT_mean_ and CIMT_max_ were 0.679 and 0.743. **D)** US image captured using 85 dB, CIMT_mean_ and CIMT_max_ were 0.691 and 0.750 millimetre.

The distal two centimetres of the CCAs were depicted in the longitudinal plane, with the diverging of the artery walls towards the carotid bifurcation as the distal limitation. The intima- and media layers were visible in both the near and the far wall. The images were captured in the end diastolic phase (ECG-assisted), when the artery had its smallest diameter. The image plane was most often an oblique sagittal plane, however, depending on the arteries tortuousness and depth, the image plane was shifted towards the coronal plane to better visualize the arteries parallel to the skin surface. The end-diastolic frame of the third cardiac cycle was chosen to explore the effect of DR changes on CIMT measures. For this frame the DR setting was changed in 15 dB steps from 40 dB to 85 dB, and images for the different DR settings were obtained. All other imaging parameters were kept fixed. Thus, four identical images, apart from the DR setting, were acquired from each artery (Figure 2). The examinations were saved in the DICOM format and exported to a remote computer for measurements of the CIMT.

### CIMT measurements

The commercially available semi-automatic intima-media thickness measurement software Máth 3.2.0 (Intelligence in Medical Imaging, Paris, France) was used for CIMT measurements. The operator identified the area to measure: a continuous one-centimetre segment of the distal two centimetres in the CCA far wall. The software automatically detected the intima-lumen and the media-adventitia interfaces and calculated average CIMT (CIMT_mean_), maximum CIMT (CIMT_max_) and the local variation of CIMT in the segment (CIMT_sd_) for DR of 40, 55, 70 and 85 dB, respectively. The coefficient of variance (CV) was calculated for repeated measurements in 10 randomly selected participants, for both CIMTmean and CIMTmax. The CV and the corresponding 95% confidence interval (95% CI) was calculated for 10 repeated measurements for 40, 55, 70 and 85 dB.

### Data analyses

The statistical analysis was done in SPSS (IBM SPSS Statistics 21, Armonk, NY, USA). Descriptive statistics was used to describe the study populations. Groups of normally distributed data were compared using the two-way student t-test and the paired student *t*-tests.

## Results

A total of 313 CCAs were included in the analysis: 181 CCAs from participants in the ABD-study and 132 CCAs from participants in the INFO-study. Population mean CIMT_mean_ was 0.529, 0.564, 0.590 and 0.605 mm with DR of 40, 65, 70 and 85 dB, respectively. Corresponding results for CIMT_max_ was 0.626, 0.667, 0.698 and 0.716. Differences between the DR steps were all statistically significant (p < 0.01) (Tables 2 and 3). The relative increases in CIMT_mean_ between DR steps were equal to the relative increase in CIMT_max_ for the same steps (p > 0.44). The relative increases for CIMT_mean_ and CIMT_max_ were 6.7% and 7.0% for 40–55 dB, 4.9% and 4.8% for 55–70 dB, and 2.6% and 2.7% for 70–85 dB, respectively.

**Table 2:** **Measurements of CIMT with different DR settings**

Population	CIMT measure	n	DR setting			
			40 dB	55 dB	70 dB	85 dB
**ABD**	CIMT_mean_ [mm]	181	0.488 ± 0.066	0.521 ± 0.066	0.547 ± 0.066	0.563 ± 0.066
	CIMT_max_ [mm]	181	0.578 ± 0.074	0.619 ± 0.072	0.648 ± 0.071	0.669 ± 0.070
**INFO**	CIMT_mean_ [mm]	130	0.586 ± 0.091	0.621 ± 0.093	0.648 ± 0.095	0.662 ± 0.095
	CIMT_max_ [mm]	130	0.691 ± 0.107	0.732 ± 0.107	0.765 ± 0.109	0.780 ± 0.108
**Total**	CIMT_mean_ [mm]	311	0.529 ± 0.091	0.564 ± 0.093	0.590 ± 0.094	0.605 ± 0.093
	CIMT_max_ [mm]	311	0.626 ± 0.105	0.667 ± 0.105	0.698 ± 0.106	0.716 ± 0.104

Data on DR 40 dB was missing in one participant in the ABD group and two in the INFO group.

All measurement values are given as mean and standard deviation.

**Table 3:** **Absolute differences in CIMT between the different DR steps**

	DR step	ABD		INFO		TOTAL	
		Difference	95% CI	Difference	95% CI	Difference	95% CI
	[dB]	[mm]	[mm]	[mm]	[mm]	[mm]	[mm]
**CIMT**_**mean**_	40-55	0.032	(0.030, 0.035)	0.037	(0.034, 0.041)	0.034	(0.032, 0.036)
	55-70	0.026	(0.025, 0.028)	0.027	(0.025, 0.030)	0.027	(0.025, 0.028)
	70-85	0.016	(0.014, 0.017)	0.014	(0.012, 0,016)	0.015	(0.014,0.016)
**CIMT**_**max**_	40-55	0.041	(0.037,0.044)	0.043	(0.039, 0.048)	0.042	(0.039, 0.045)
	55-70	0.029	(0.026,0.032)	0.033	(0.029, 0.038)	0.031	(0.029, 0.033)
	70-85	0.020	(0.018,0.023)	0.015	(0.012, 0.019)	0.018	(0.016, 0.020)

For all comparisons p < 0.001. All differences are given as mean values, and in millimetres.

Average change in CIMT_mean_ from 40 to 85 dB was 0.076 mm corresponding to approximately 0.008 mm for each 5 dB increase. For all measurements mean CIMT_sd_ was less than 0.05 mm.

The CV for CIMT_mean_ was 0.65% (95% CI; 0.48-0.83) for 40 dB, 0.42% (95% CI; 0.28-0.56) for 55 dB, 0.86% (95% CI; 0.51-1.21) for 70 dB and 0.59% (95% CI; 0.37-0.82) for 85 dB. For CIMT_max_ the CVs were 0.59% (95% CI; 0.33-0.85), 0.85% (95% CI; 0.16-1.36), 1.36 (95%CI; 0.37-2.37) and 1.04% (95% CI; 0.41-1.67) for 40, 55, 70 and 85 dB, respectively.

## Discussion

This study showed that an increase in DR in US image acquisition yields increased CIMT values and that the effect of increasing DR is largest for lower DRs. The DR should therefore be specified in all US protocols where CIMT values are used either directly or as a predictive biomarker, especially in longitudinal follow-up assessments.

As DR increases the range of signal intensities included in the image formation increases. This increase yields reduced image contrast and less defined interfaces between the intima, media and adventitia layers in the artery wall. Consequently, it is anticipated that CIMT would increase with increasing DR. In the interval between 40 and 85 dB the average increase in CIMT_mean_ per 5 dB increase in DR was 0.008 mm. This is approximately 2.5 times higher than the values found by Potter et al. in a phantom study [10]. The phantoms were made of tissue-mimicking agar with wall thicknesses of 0.5, 1.0 and 1.5 mm and acoustic properties similar to tissue. In our study, the changes in CIMT measurements due to DR variation were anticipated to be in the same range as the phantom with 0.5 mm wall thickness. The discrepancy in findings may be related to the composition of the wall (phantom versus human arteries). In contrast to human arteries, the phantom walls were homogenous with defined interfaces and consequently, interface echo signals will be more distinct in phantoms than in human arteries. The relative change in CIMT_mean_ for CCAs was largest between 40 and 55 dB (6.7%) and smallest between 70 and 85 dB (2.6%) indicating a declining dependency for increasing DR. The relative increase found by Potter et al. for DR of 40 to 60 dB was about 3% and as such our findings suggest an underestimation of DR dependency in phantoms, especially for low DRs. The algorithms used for the automatic edge detection as well as larger variation in the tissue composition of human arteries than in the phantoms might have contributed to the differences between the studies. We speculate that the variation in the human tissue causes a broader spectrum of echo signals, and that the effect of widening the DR therefore will be more prominent in this setting. However, despite differences in magnitude both Potter et al. and we found that CIMT increased for increasing DR.

The CIMT_mean_ and CIMT_max_ found for different DRs in our study emphasize the need for reporting DR used in US protocols as CIMT and change in CIMT over time are used as tools for risk-stratification, as surrogate end-points, and as references for clinical decision-making: European Guidelines on cardiovascular disease prevention in clinical practise 2012 states that CIMT > 0.9 mm is considered abnormal [11]. The extreme DR values (40 and 85 dB) in our study yielded a difference in CIMT_mean_ and CIMT_max_ of 8-10% of the suggested reference value. Knowledge of DR setting is important for patients where CIMT values are close to cut-off values or reference values of clinical importance. Different DR between baseline and follow-up examinations may lead to under- or overestimation of CIMT change. Outcome of multicentre studies may also be compromised. Thus, by including information of DR in guidelines, reference data and study protocols will increase the validity of the results.

A shortcoming of our study is the large DR steps (15 dB). Smaller DR steps would probably give more precise results of the relation between measured CIMT and DR setting. However, the wide range of DRs included allowed identification of the non-linearity between change in CIMT and change in DR that would probably not have been detected if DR had been restricted to 40 to 60 dB, as in Potter et al. [10]. The large number of participants and the fact that the relative CIMT changes for different DRs were similar for the two study populations differing in age and gender mixture strengthens the external validity of our findings.

## Conclusions

In conclusion, DR significantly changes CIMT measurements and the changes are most prominent for lower DRs. The effect of changing DR is larger in human arteries than in phantoms. Reporting the DR will therefore increase the validity of CIMT data, especially in prospective clinical trials.

## Abbreviations

CCA: Common carotid artery
CIMT: Carotid intima media thickness
DR: Dynamic range
US: Ultrasound
